# Griffiths phases and localization in hierarchical modular networks

**DOI:** 10.1038/srep14451

**Published:** 2015-09-24

**Authors:** Géza Ódor, Ronald Dickman, Gergely Ódor

**Affiliations:** 1 MTA-MFA-EK Research Institute for Technical Physics and Materials Science, H-1121 Budapest, P.O. Box 49, Hungary; 2 Departamento de Fisica and National Institute of Science and Technology of Complex Systems, ICEx, Universidade Federal de Minas Gerais, Caixa Postal 702, 30161-970, Belo Horizonte - Minas Gerais, Brazil; 3 Massachusetts Institute of Technology, 77 Massachusetts Avenue Cambridge, MA 02139-4307, USA

## Abstract

We study variants of hierarchical modular network models suggested by Kaiser and Hilgetag [ *Front. in Neuroinform.,* **4** (2010) 8] to model functional brain connectivity, using extensive simulations and quenched mean-field theory (QMF), focusing on structures with a connection probability that decays exponentially with the level index. Such networks can be embedded in two-dimensional Euclidean space. We explore the dynamic behavior of the contact process (CP) and threshold models on networks of this kind, including hierarchical trees. While in the small-world networks originally proposed to model brain connectivity, the topological heterogeneities are not strong enough to induce deviations from mean-field behavior, we show that a Griffiths phase can emerge under reduced connection probabilities, approaching the percolation threshold. In this case the topological dimension of the networks is finite, and extended regions of bursty, power-law dynamics are observed. Localization in the steady state is also shown via QMF. We investigate the effects of link asymmetry and coupling disorder, and show that localization can occur even in small-world networks with high connectivity in case of link disorder.

In neuroscience, the criticality hypothesis asserts that the brain is in a critical state, at the boundary between sustained activity and an inactive regime. Theoretical and experimental studies show that critical systems exhibit optimal computational properties, suggesting why criticality may have been selected in the evolution of the nervous system^1^. Although criticality has been observed in cell cultures^2^,^3^, brain slices and anesthetized animals^4^,^5^, the debate regarding criticality in alert animals and humans continues^6^,^7^,^8^. Thus the criticality hypothesis remains controversial in brain science; for a review see^9^.

Normally, for a system to be at criticality, certain control parameters need to be tuned precisely, raising the perennial question of how such tuning is achieved in the absence of outside intervention. The possibility of self-tuning is well known in statistical physics; the paradigm of self-organized criticality (SOC) has been studied since the pioneering work of^10^. Simple homogeneous models such as the stochastic sandpile exhibit criticality with power laws both in statics and dynamics. This has been understood as the result of a control mechanism that forces the system to an absorbing-state phase transition^11^.

Real nervous systems, however, are highly inhomogeneous, so that one must take into account the effects of heterogeneities. It is well known in statistical physics that disorder can cause rare-region (RR) effects^12^ that smear the phase transitions. These effects can make a discontinuous transition continuous, or generate so-called Griffiths phases (GP)^13^, in which critical-like power-law dynamics appears over an extended region around the critical point. In these regions, moreover, non-Markovian, bursty behavior can emerge as a consequence of a diverging correlation time. The inter-communication times of the nodes (which possess no internal memory) follow a fat-tailed distribution^14^. Thus, heterogeneities widen the critical region and can contribute to the occurrence of power laws. This provides an alternative mechanism for critical-like behavior without fine tuning, although attaining the GP does require some rough tuning, of the sort that is not difficult to find in biological systems.

It was shown recently that the topological heterogeneity of the underlying networks can result in GPs in finite-dimensional systems^15^ and can be a reason for the working memory in the brain^16^. Although many networks exhibit the small-world property and so have an infinite topological dimension, naturally occurring networks are always finite, exhibit cutoffs, and therefore GPs can be expected as a consequence of inhomogeneous topology^17^.

Many real networks can be partitioned seamlessly into a collection of modules. Each module is expected to perform an identifiable task, separate from the function of others^18^. It is believed that the brain is organized hierarchically into modular networks across many scales, starting from cellular circuits and cortical columns via nuclei or cortical areas to large-scale units such as visual or sensory-motor cortex. At each level, connections within modules are denser than between different modules^19^,^20^,^21^,^22^. Although empirical data confirm this modular organization on some scales^23^, the detailed organization of brain networks is not yet experimentally accessible.

Two particular kinds of hierarchical modular networks (HMN-1,HMN-2) model were proposed and investigated numerically and analytically in^24^. On large-world HMNs, which imply a finite topological dimension *D*, models of the spread of activity exhibit power-law dynamics and rare region effects. However, these power laws are system-size dependent, so that true GP behavior has not yet been proven. These authors also simulated spreading on empirical brain networks, such as the human connectome and the nervous system of *C. elegans*. Available empirical networks are much smaller than the synthetic ones and deviations from power laws are clearly visible. Both anatomical connections^19^ and the synchronization networks of cortical neurons^25^ suggest small-world topology^26^. The brain network modules of^24^ are weakly coupled in such a way that these HMNs are near the percolation threshold, as in the case of the models introduced in^15^. Note, however that requiring the network to be near percolation again raises tuning and robustness issues. Having weaker ties would lead to fragmented networks, while stronger ties result in infinite *D* and the absence of a GP. In the present work we do not assume such fine tuning: we maintain a high density of short edges, rendering the networks well connected. Another way of preserving the integrity of HMN networks with finite dimension involves the random tree-like structures studied here.

To study synchronization^27^, commonly expected in brain dynamics, the Kuramoto model^28^ has been implemented^29^ on the same networks as studied in^24^. In this case even weaker rare-region effects were found, resulting in stretched exponential dynamics. One of the main purposes of our study is to delineate conditions a GP. While the networks studied are of finite size, repeating the process on many network realizations and averaging over them, we clearly see convergence towards GP dynamics. We assume that multiple random network realizations may occur in the brain over time, due to reconfigurations of the synapses or as a consequence of weakly coupled sub-modules and changes in overall intensity of connections between different brain regions due to neuromodulation.

In addition to the dimension, other topological features, which have yet to be studied systematically, may also influence RR effects. The clustering coefficient, defined as


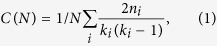


where *n*_*i*_ denotes the number of direct links interconnecting the *k*_*i*_ nearest neighbors of node *i*, exhibits different scaling behavior in modular networks than in unstructured networks. While in SF networks it decays as *C*(*N*) ~ *N*^−3/4^ and in random networks as *C*(*N*) ~ *N*^−1^, in HMNs it is constant. Furthermore, while in SF networks the clustering is degree-number independent, in HMNs it decays as *C*(*k*) ~ *N*^−*β*^, with *β* in the range 0.75–1^30^. This means that the higher the node degree, the smaller is its clustering coefficient, possibly reducing its infectiousness in an epidemic process. This suggests an enhancement of localization as in SF models with disassortative weighting schemes^31^.

In this work we study spreading models defined on HMNs embedded in 2D space, and investigate how various inhomogeneities (topological or intrinsic) contribute to the occurrence of localization and GPs. We begin by considering purely topological heterogeneity; later we study cases with disorder in the connections as well. It is well known that intrinsic link disorder can appear as the consequence of asymmetry, connecting nodes in the cortex (see^32^). It has also been demonstrated that weights can vary over 4–6 orders of magnitude; most (including the majority of long-range connections) are actually quite weak^33^.

The relation of RR effects to localization in the steady state has been studied recently^17^. Here we discuss localization results obtained via a quenched mean-field (QMF) approximation.

In addition to serving as models of brain connectivity, hierarchical modular networks are abundant in nature. They occur in social relations^34^, in the WWW^35^, metabolic^36^ and information systems^37^ for example. Thus the results reported here should find application in these and related areas.

## Hierarchical modular networks

Recent studies indicate that activity in brain networks persists between the extremes of rapid extinction and global excitation. This so-called limited sustained activity (LSA) does not occur in spreading models defined on structureless, small-world networks. On the other hand, brain networks are known to exhibit hierarchical modular structure. This motivated Kaiser and Hilgetag (KH) to perform numerical studies on such networks, to investigate topological effects on LSA^38^. Their hierarchical model reflects general features of brain connectivity on large and mesoscopic scales. Nodes in the model were intended to represent cortical columns rather than individual neurons. The connections between them were taken as excitatory, since there appear to be no long-distance inhibitory connections within the cerebral cortex^39^.

Kaiser and Hilgetag generated networks beginning at the highest level, and adding modules to the next lower level, with random connectivity within modules. They explored hierarchical networks with different numbers of levels, and numbers of sub-modules at each level. The average degree (over all nodes in the network) was set to 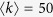, motivated by experimental studies. They investigated different topologies by varying the edge density across the levels. All the networks studied by KH are of small-world type, i.e., they have an infinite topological dimension.

The spreading model investigated by KH is a two-state threshold model, in which, at each time step, inactive nodes are activated with probability *λ* if at least *m* of their neighbors are currently active, and active nodes deactivate spontaneously with probability *v*. This model is very similar to reaction-diffusion models known in statistical physics^40^,^41^,^42^, with a synchronous cellular automaton (SCA) updates. Starting from an initial state in which a localized set of nodes is active, these authors followed the density of active sites and their localization up to 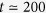 time steps on networks of sizes *N* ≤ 512.

Kaiser and Hilgetag found that LSA can be found in a larger parameter range in HMNs, as compared with random and non-hierarchical small-world networks. The optimal range of LSA was found in networks in which the edge density increased from the top level (*l* = *l*_*max*_) to the bottom (*l* = *l*_1_). Such topologies foster activity localization and rare-region effects.

In this paper we investigate HMNs which possess increasing edge density from top to bottom levels, as did KH, but with *finite* topological dimension. We will show that although localization is seen in small world networks, to observe GPs, with power-law dynamics, we need networks of finite topological dimension.

**Figure 1:**
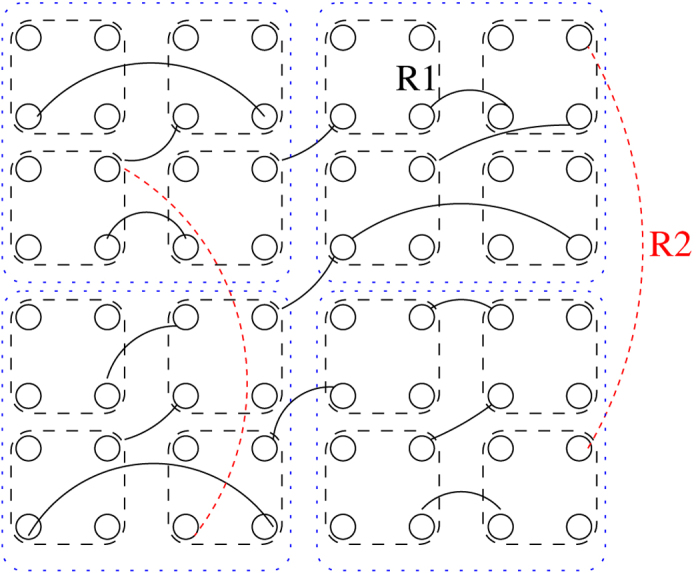
Two lowest levels of the HMN2d hierarchical network construction. Dashed lines frame bottom level nodes, which are fully connected there, dotted lines frame the nodes of the next level. The solid lines denoted R1 are randomly chosen connections among the bottom level modules, ensuring single connectedness of the network, while those denoted R2 provide random connections on the next level. Links can be directed.

To generate a hierarchical modular network, we define *l*_*max*_ levels on the same set of 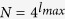 nodes; on the *l*^*th*^ level we define 4^*l*^ modules. We achieve this by splitting each module into four equal sized modules on the next level, as if they were embedded in a regular, two-dimensional (2d) lattice (see Fig. 1). The probability *p*_*l*_ that two nodes are connected by an edge follows 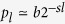 as in^38^, where *l* is the greatest integer such that the two nodes are in the same module on level *l*. After selecting the number of levels and nodes we fix the average node degree, 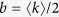, and generate the adjacency matrix *A* by proceeding from the highest to the lowest level. We fill the submatrices with zeros and ones and copy them to appropriate diagonal locations of *A*. We allow unidirectional connections, which is more realistic. In fact, disorder associated with the link orientations turns out to be necessary to observe GPs. Finally, we connect the lowest-level modules with an edge, chosen randomly from nodes of *l*_1_. This provides a short-linked base lattice that guarantees connectivity.

In a preliminary study, the extra edges, corresponding to the base lattice, were not added. The resulting networks typically consist of a large number of isolated connected components; the GP effects observed in these structures are a consequence of fragmented domains of limited size. Measurements of axon-length distributions in several real neural networks show a large peak at short distances followed by a long flat tail. Thus there is a dense set of local edges in addition to a sparse network of long-range connections, best fit by an exponential function^43^. Typical estimates indicate that 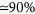 of all cortical connections are formed at the local level (i.e., within a radius of 0.5mm); only the remainder leave the local gray matter and project to remote targets. Therefore, we also investigate a variant of HMN2d networks in which the lowest-level modules are fully connected, and there is a single link among the nodes of modules on level *l*_2_. To broaden further the range of structures, we study *hierarchical modular trees* (HMTs), which possess the minimum number of edges required for connectivity, and so have no loops. Construction of HMTs is described in the Supplementary material.

### Relation to Benjamini-Berger networks

Due to the embedding, there is a correspondence with Benjamini-Berger (BB) networks^44^. BB networks are generated on Euclidean lattices, with short links connecting nearest neighbors. In addition, the network contains long links, whose probability decays algebraically with Euclidean distance *R*:


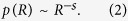


Here we consider modified BB networks, in which the long links are added level by level, from top to bottom, as in^38^. The levels: 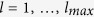 are numbered from the bottom to top. The size of domains, i.e., the number of nodes in a level, grows as *N*_*l*_ = 4^*l*^ in the 4-module construction, related to a tiling of the two dimensional base lattice. Due to the approximate distance-level relation, 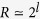, the long-link connection probability on level *l* is:


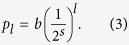


Here *b* is related to the average degree of a node 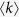.

It is known that in a one-dimensional base lattice the BB construction results in a marginal case, *s* = 2, in which the topological dimension is finite and changes continuously with *b*. For *s* < 2 we have small world networks, while for *s* > 2 the the topological dimension is the same as the base lattice (*d* = 1) in BB networks. The HMN-1 networks studied by Moretti and Muñoz^24^ are similar to the BB model, without the 1d base lattice, but with the inclusion of a HMN topology. These authors simulated spreading models on HMN-1 with finite topological dimension at 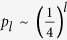 and found GPs. Given the above mapping, this result is not very surprising, because this HMN corresponds to the *s* = 2 case, staying close to the percolation threshold of the network. To ensure connectivity, Moretti and Muñoz added extra links to the large connected components^24^; they assert that the topological dimension remains finite, despite the additional links. In networks with finite sizes this is certainly true, however in the infinite size limit such a system is also infinite dimensional, so that we don’t expect true GPs. For the HMN-2, the number of connections between blocks at every level is a priori set to a constant value. In this case numerics of^24^,^45^ suggest GP effects.

Here we embed the HMNs in 2d base lattices, which is closer to the real topology of cortical networks. In this case the threshold for marginality (i.e., continuously changing dimension and exponents) is expected to be at *s* = 4. We confirm this by explicit measurements of the topological dimension. Furthermore, we study two-dimensional HMNs with different *s* values and find GP effects for finite topological dimensions, both at the percolation threshold (for *s* = 3), and for *s* = 6, where the tail distribution of the link lengths decays very rapidly, in agreement with empirical results for axon lengths.

In addition to the CP (*m* = 1), we also consider threshold models (*m* ≥ 2), which are expected to be more realistic for neuronal systems.

### Dimension measurements

To measure the dimension of the network we first compute the level structure by running a breadth-first search from several, randomly selected seeds. We count the number of nodes *N*(*r*) with chemical distance *r* or less from the seeds and calculate averages over the trials. We determined the dimension of the network from the scaling relation *N* ~ *r*^*d*^, by fitting a power-law to the data for *N*(*r*).

**Figure 2:**
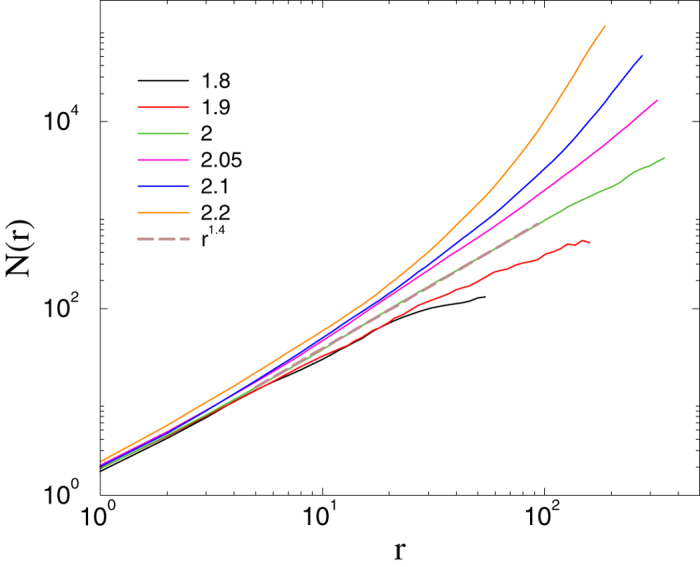
Number of nodes within the chemical distance *r* in the *s* = 3 HMN2d models with *l*_*max*_ = 9. Different curves correspond to different parameters: 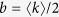 as indicated. The dashed line shows a power-law fit for *b* = 2, with *N*(*r*) ~ *r*^1.4^, suggesting a topological dimension of *d* = 1.4 at the percolation threshold.

**Figure 3:**
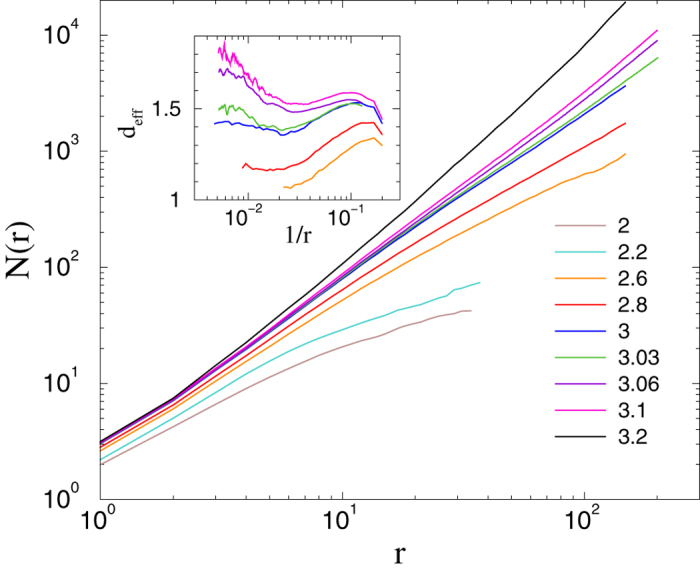
Number of nodes within chemical distance *r* in HMN2d networks with *s* = 4 and *l* = 9 levels. Different curves correspond to different 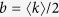 values as indicated. Inset: local slopes *d*_*eff*_ of the *N*(*r*) curves, defined in equation (4).

At *s* = 3 we observe a percolation threshold near 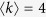, for which the topological dimension is finite: 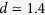 (see Fig. 2). Note that the curves with large 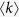 exhibit saturation, corresponding to a finite-size effect. The case *s* = 4 appears to correspond to a marginal dimension, for which, above the percolation threshold 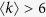, one observes a continuously varying topological dimension (see Fig. 3). A more detailed, local slopes analysis via the effective topological dimension


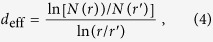


is shown in the inset of Fig. 3, where *r* and *r*′ are neighboring measurement points. Saturation to *s*-dependent dimensions can be read off the plot for *r* > 100.

**Figure 4:**
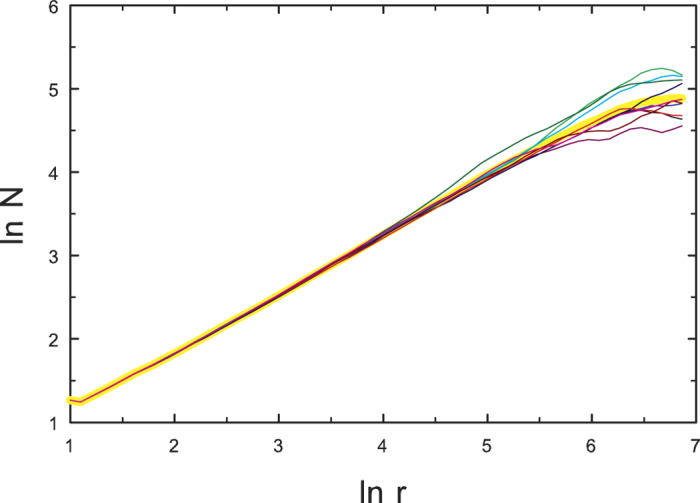
Number of nodes within chemical distance *r* in a set of ten random hierarchical trees with 262144 nodes (thin curves). The broad yellow curve is an average over the set of ten structures.

**Figure 5:**
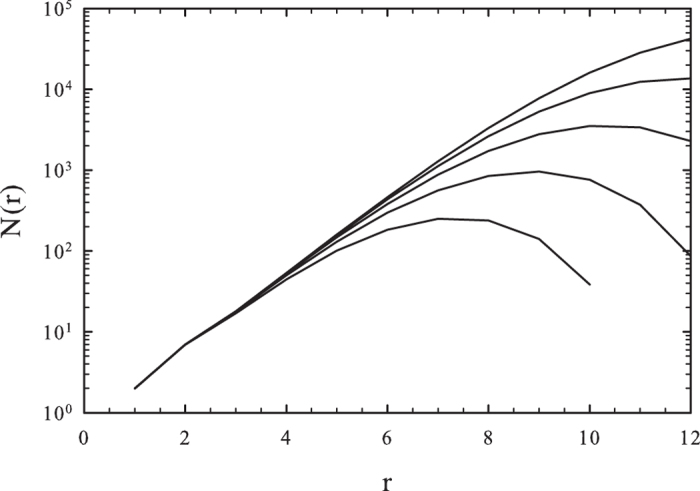
Number of nodes within chemical distance *r* in regular hierarchical trees with 1024, 4096, …, 262144 nodes (lower to upper curves).

Random hierarchical trees also exhibit a finite topological dimension. Fig. 4 shows *N*(*r*) for a set of ten independent random trees. The average of *N*(*r*) over the set of ten trees follows a power law to good approximation, with 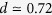. (Note that in this case *N*(*r*) is an average over *all* nodes.) For regular trees, by contrast, *N*(*r*) grows exponentially with *r*, as shown in Fig. 5. Thus the regular tree construction results in a structure with infinite topological dimension.

### Dynamic simulations

#### Contact process

The *contact process* (CP)^46^,^47^, a Markov process defined on a network, can be interpreted as a simple model of information spreading in social^48^, epidemic spreading^49^,^50^,^51^, or in brain networks^24^. In the CP sites can be active or inactive. Active sites propagate the activity to their neighbors at rate *λ*/*k*, or spontaneously deactivate with rate *v* = 1 (for simplicity).

**Figure 6:**
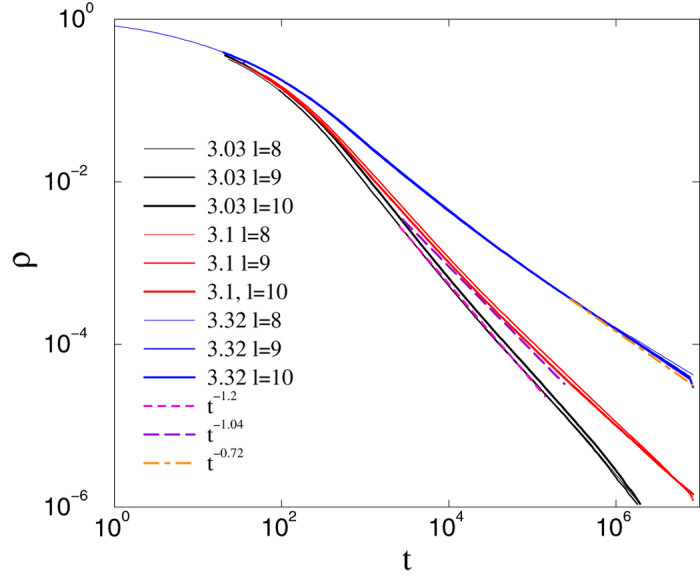
CP on asymmetric HMN2d networks with *s* = 4: decay of activity for *λ* values as indicated. System sizes *l*_*max*_ = 8, 9, 10 (thin, medium, and thick lines, respectively). Size-independent power laws are evidence of a Griffiths phase.

We perform extensive activity-decay simulations for HMN2d models with 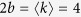 and maximum levels: *l*_*max*_ = 8, 9, 10. In these networks we use directed links between nodes, similar to real nervous systems. We follow *ρ*(*t*) in 10–100 runs for each independent random network sample, starting from fully active initial states, and averaging *ρ*(*t*) over thousands of independent random network samples for each *λ*. In the marginal long-link decay case at *s* = 4, a clear GP behavior is found (see Fig. 6).

**Figure 7:**
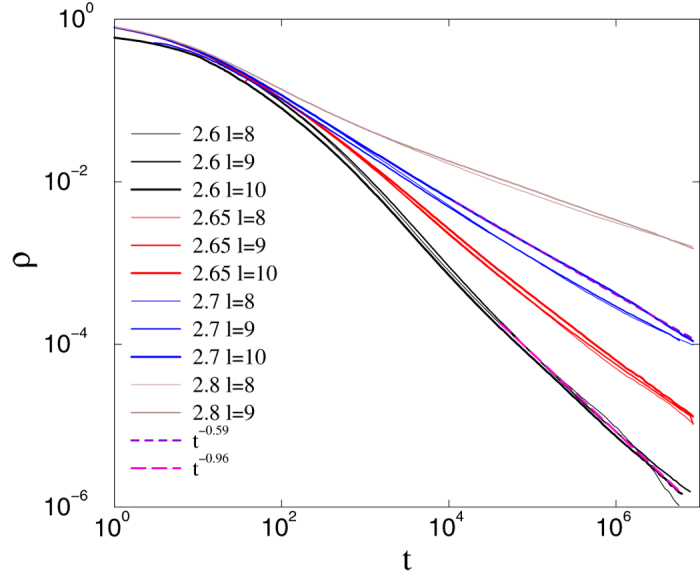
CP on asymmetric HMN2d networks as in Fig. 6, but for *s* = 3.

We show that a GP can occur for more general parameters than those studied in^24^, by following the density decay in networks with *s* = 3 and 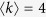 (i.e., at the percolation threshold). Size-independent, nonuniversal power laws can be seen in Fig. 7.

When we increase the average degree 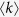, the GP shrinks to a smaller range of *λ* values, as in^15^, tending toward a simple critical phase transition. However, it is hard to determine at precisely which value of 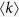 this happens. We note that the connectivity of the network is not assured, without the addition of extra links. Strictly speaking, however, with this extension *s* ≤ 4 networks become infinite-dimensional in the 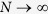 limit.

**Figure 8:**
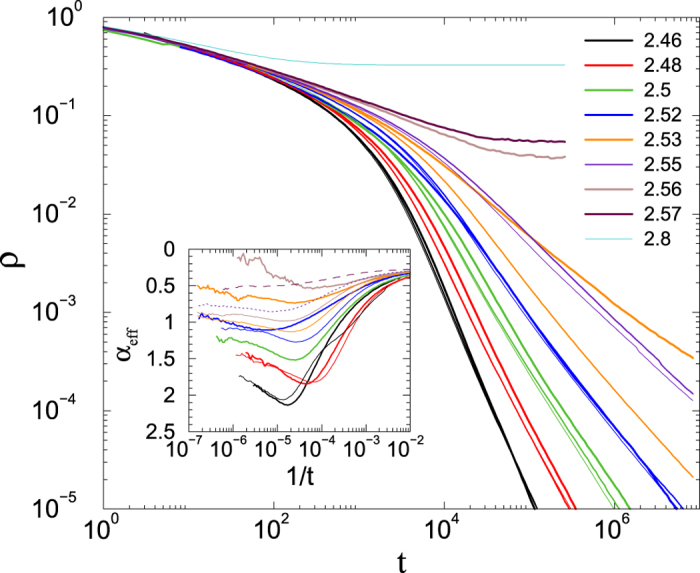
CP on asymmetric HMN2d networks with *s* = 6 and 〈*k*〉 ≃ 4: decay of activity for *λ* values as indicated. System sizes as in Fig. 6. Size-independent power laws are observed for 2.45 < *λ* < 2.53. Inset: local slopes of the curves in the main plot. In the GP *α*_*eff*_ tends to nonuniversal values with logarithmic corrections. At *λ*_*c*_ = 2.53(1) we observe convergence to *α*_*eff*_ = 1; above this threshold the effective exponents of larger systems veer upwards toward zero as *t* tends to infinity.

More importantly, GPs are also found in networks connected on the base level via short edges. We study the activity decay for *s* = 6, which corresponds to fast decaying tail distribution for the long links, preserving finite topological dimension *d*. In this construction the average degree at the bottom level is 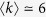, decreasing systematically with *l*, so that 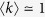 for *l*_*max*_. Note that that the ratio of short to long links is ~0.11, in agreement with results for real neural networks^43^. Simulations again yield size-independent power-law decay curves, confirming GP behavior, as shown in Fig. 8.

We also determined the effective decay exponent in the usual manner (see^42^), via


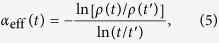


where *ρ*(*t*) and 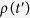 are neighboring data points. The critical point can be located at *λ*_*c*_ = 2.53(1), showing mean-field scaling: *ρ*(*t*) ~ *t*^−1^. Above this threshold power-laws can still be seen for smaller sizes (*l*_*max*_ = 8, 9), up to 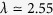, but corrections to scaling become stronger; the curves for *l*_*max*_ = 10 exhibit saturation. This is surprising, because in a disordered system with short ranged interactions one would expect a critical phase transition with an ultra-slow, logarithmic scaling. However, recent studies of the CP in higher dimensions find mean-field criticality and GP^52^. Our result suggests that the topological heterogeneity generated by the long edges is not strong enough to induce an infinite-randomness fixed point. Otherwise we must assume very strong corrections to scaling in this case.

Disorder due to the randomly chosen orientations of the links turns out to be relevant. For *symmetric* links our simulations yield nonuniversal power laws, which appear to be sensitive to the system size, suggesting the lack of a true GP in the infinite-size limit.

#### Burstyness in the CP

**Figure 9:**
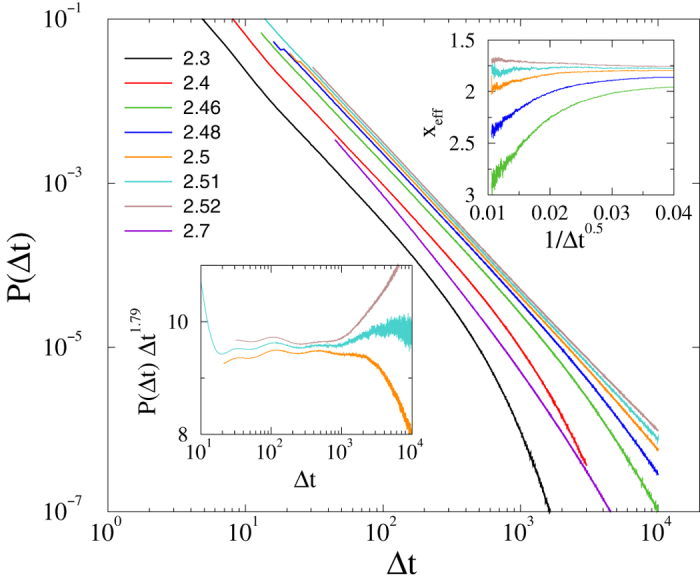
CP on asymmetric HMN2d networks with *s* = 6. Main plot : probability distribution, *P*(Δ*t*), of inter-event times for *λ* values as indicated; system size *l*_*max*_ = 10. Power-law tails are evident for 2.46 < *λ* < 2.6, with continuously changing exponents. Right inset: local slopes of the same curves, defined similarly as in equation (5) for: *λ* = 2.46, 2.48, 2.5, 2.52, 2.7. Left inset: 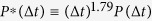 for *λ* = 2.5, 2.51, 2.52.

We study the distribution of inter-event times in the CP on asymmetric, HMN2d networks with *s* = 6, in a manner similar to that described in^14^. Starting from fully active states, on networks of size *l*_*max*_ = 10, we measured 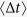 between successive activation attempts. We followed the evolution up to *t*_*max*_ = 2^18^ Monte Carlo steps (MCs), averaging over 1000–2000 independent random networks with 10–100 runs for each. Througout this paper time is measured by MCs. As Fig. 9 shows, fat-tailed distributions, 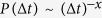, emerge in the GP for 2.46 < *λ* < 2.6, while *P*(Δ*t*) decays exponentially outside this region. The slopes of the decay curves are determined via least-squares fits in the window 20 < Δ*t* < 7000. For *λ* = 2.5 we find *x* = 1.753(4), while for *λ* = 2.52 the exponent is slightly larger: *x* = 1.81(1). These values are close to the auto-correlation exponent of the critical 1 + 1 dimensional CP as in^14^, but exhibit deviations due to the heterogeneities. This is understandable, since the effective dimension of this HMN2d is close to one. The scaling variable 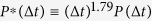 exhibits log-periodic oscillations. This is the consequence of the modular structure of the network. Furthermore, as in other GP models, logarithmic corrections to scaling are expected.

We expect similar nonuniversal, control parameter dependent tails in *P*(Δ*t*) in the GPs exhibited by the other HMN2d networks. Furthermore, as shown in^14^, power-law distributions should arise for other initial conditions, such as localized activity. This suggests that bursty inter-communication events in brain dynamics arise spontaneously near the critical point, in the GP.

#### CP on random hierarchical trees

We simulate the CP with *symmetric* links on random hierarchical trees (RHTs) of 262144 nodes. We first perform quasistationary (QS) simulations^53^, of the CP on a *single* RHT. For one structure this yields 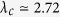; for another, independently generated structure of the same kind, we find 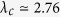.

**Figure 10:**
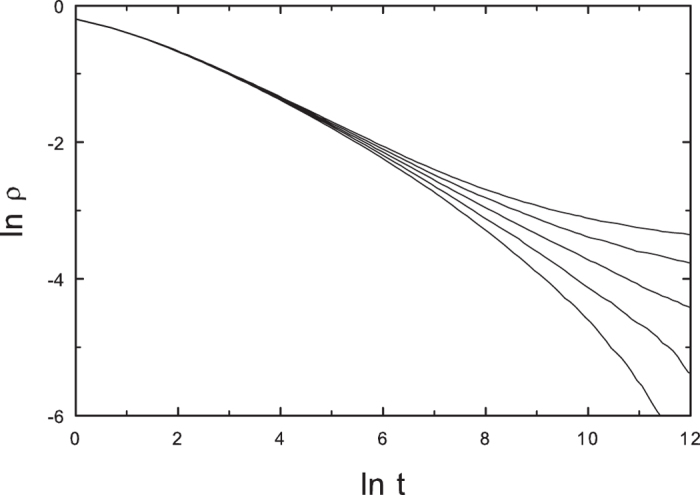
CP on a random hierarchical tree: activity versus time for *λ* = 2.73, 2.74, …, 2.77 (lower to upper); system size: *N* = 262144. Power-law decay is evident only for *λ* = 2.75, close to the estimate *λ*_*c*_ = 2.76 derived from QS simulations. Each curve represents an average over 100 realizations; all realizations are performed on the same network.

Our principal interest is in the decay of activity starting from all nodes active. The decay of activity on a *single* RHT appears to follow the scenario familiar from the CP on regular lattices: power-law decay is observed at *λ*_*c*_ but not at nearby values, as illustrated in Fig. 10. The randomness associated with a single RHT appears to be insufficient to generate a Griffiths phase.

**Figure 11:**
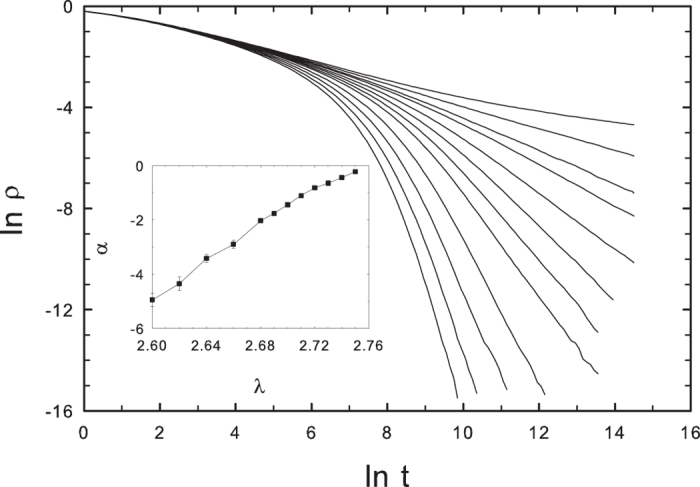
CP on a random hierarchical trees: activity versus time for *λ* = 2.60, 2.62, 2.64, 2.68, 2.69, …, 2.75 (lower to upper); system size *N* = 262144. Each curve represents an average over 10^3^–10^4^ realizations, each performed on a different network. Inset: decay exponent −*α* versus *λ*.

By contrast, evidence of a GP is found if we average over many RHTs. The activity averaged over a large set (10^3^–10^4^) of independent realizations, each on a different RHT, shown in Fig. 11, decays asymptotically as a power-law over a fairly broad range of subcritical *λ* values. The decay exponent *α* extrapolates to zero at 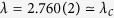, as shown in the inset. We note that the average is over *all* realizations, including those that become inactive before the maximum time of 2 × 10^6^ MCs. If we instead restrict the average of *ρ*(*t*) to trials that survive to time *t* (or greater), the result is a stretched exponential, 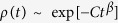, where *C* is a constant and the exponent *β* varies with *λ*. For *λ* = 2.70, for example, 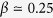.

#### Threshold model simulations

Threshold models represent an attempt to capture the activation threshold of real neurons, by requiring at least *m* active neighbors associated with incoming links to activate a node with probability *λ*. In case of active nodes spontanous deactivation occurs with probability *v* written in the reaction-diffusion model notation as:

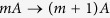
 with probability *λ*,
- with probability 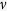.

**Figure 12:**
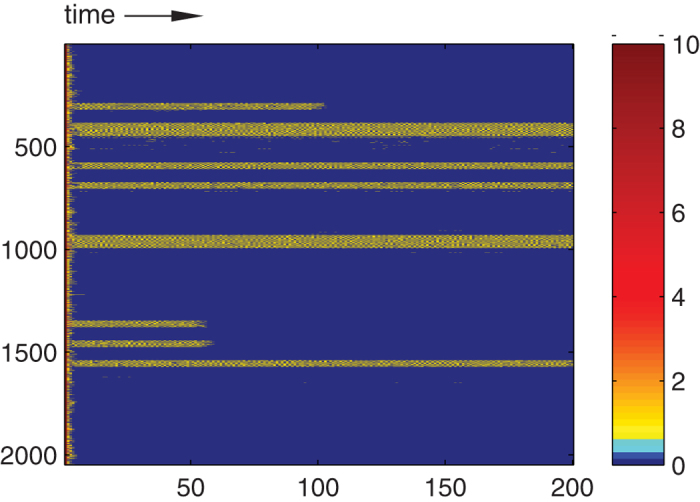
Spatio-temporal evolution of the density of the *m* = 6 threshold model, proportional to the color coding on the right. The simulation is started from an active state, with *v* = 0.9 and *λ* = 1.

We use SCA updating, as in^38^. Since there is no spatial anisotropy (which might generate activity currents), we can assume that the SCA follow the same asymptotic dynamics as the corresponding model with random sequential updating. Thanks to the synchronous updates, we can speed up the simulations by ~*n*_*p*_ times by distributing the nodes among *n*_*p*_ multiprocessor cores; by swapping the random number generation on parallel running graphics cards we obtain a further reduction of 50% decrease in the simulation times. The spatio-temporal evolution of the HMN2d network with *m* = 6, in a single network realization, starting from a fully active state, is shown in Fig. 12. After a sharp initial drop in activity, due to spontaneous deactivation of nodes with few neighbors, one observes domains (modules) on which activity survives for a long time, suggesting rare region effects.

#### Results for threshold models

We begin by discussing the *m* = 2 case, for which extensive simulations are performed. We generate networks with average degree 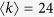 and *s* = 6. Note that in this case values of 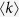 higher than the percolation threshold are needed to avoid modules having separated activities. As before, we averaged over hundreds of independent random networks and thousands of independent realizations. We followed the density up to *t*_*max*_ = 10^5^ MCs, starting from a fully active initial condition.

**Figure 13:**
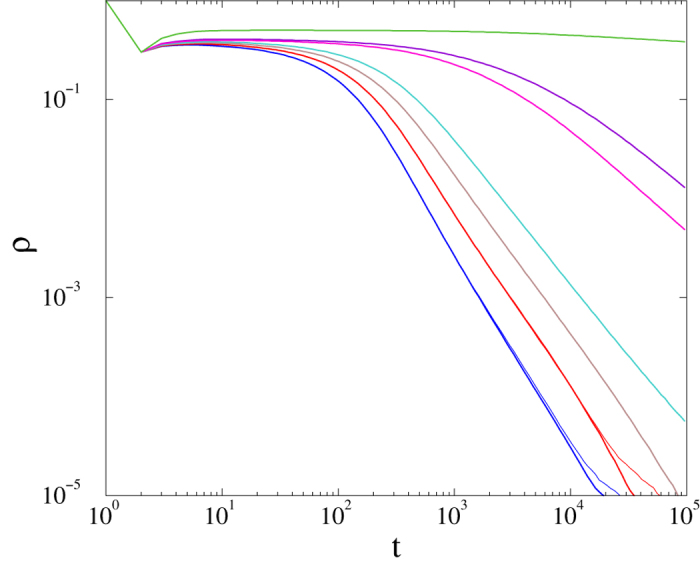
Decay of activity in the *m* = 2 threshold model with *s* = 6, and 〈*k*〉 = 24. The curves correspond to branching rates: *λ* = 0.65, 0.66, 0.67, 0.68, 0.83 (lower to upper) and *v* = 0.7 fixed. Levels: *l*_*max*_ = 8, 9 (thin, thick lines). Size-independent power-laws, reflecting a GP are observed.

In this case the mean activity density decays more rapidly than in mean-field theory, and is size-independent (see Fig. 13). For large branching probabilities, 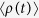 seems to take a constant value, suggesting an active steady state, but at late times some deviation is observed, possibly due to finite-size effects.

Homogeneous triplet creation models (i.e., *m* = 3) are expected to exhibit a mean-field-like *discontinuous* phase transition in two or more dimensions (see for example^54^). Disorder induces rounding effects, producing continuous phase transitions or GPs^45^,^55^. We study a threshold model with *m* = 3 and *s* = 6, using 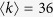. Our results are similar to those for *m* = 2: non-universal power-laws, again suggesting a GP.

### Quenched Mean-field approximation for SIS

Heterogeneous mean-field theory provides a good description of network models when fluctuations are irrelevant. This approximation is attractive because is can be solved analytically in many cases; the results agree with simulation^56^,^57^. This analysis treats nodes with different degrees as distinct, but finally averages over all degree values, providing a homogeneous solution for the order parameter. To describe the effects of quasi-static heterogeneities in a more precise way, the so-called Quenched Mean-Field (QMF) approximation was introduced^58^,^59^. For SIS models this leads to a spectral analysis of the adjacency matrix *A*_*ij*_ of the network. The susceptible-infected-susceptible (SIS)^60^ model is similar to the CP, except that branching rates are not normalized by *k*, leading to symmetric governing equations. A rate equation for the SIS model can be set up for the vector of activity probabilities *ρ*_*i*_(*t*) of node *i* at time *t*:


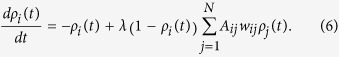


Here the *w*_*ij*_ = *w*_*ji*_ are weights attributed to the edges. For large times the SIS model evolves to a steady state, with an order parameter 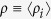. Since this equation is symmetric under the exchange 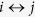, a spectral decomposition can be performed on a basis of orthonormal eigenvectors (*y*).


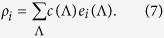


Non-negativity of the matrix 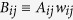 assures a unique, real, non-negative largest eigenvalue *y*_*M*_.

In the long-time limit the system evolves into a steady state and we can express the solution via *B*_*ij*_ as


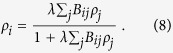


Stability analysis shows that 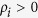 above a threshold *λ*_*c*_, with an order parameter 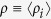. In the eigenvector basis equation (8) can be expanded by the coefficients *c*(Λ) as


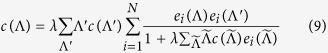


and near the threshold we can express 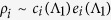 with the principal eigenvector. In the QMF approximation 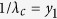 and the order parameter can be approximated by the eigenvectors of the largest eigenvalues


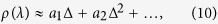


where 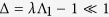 with the coefficients


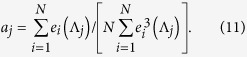


As proposed in^59^, and tested on several SIS network models^17^, localization in the active steady state can be quantified by calculating the inverse participation ratio (IPR) of the principal eigenvector, related to the eigenvector of the largest eigenvalue *e*(*y*_1_) of the adjacency matrix as


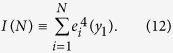


This quantity vanishes ~1/*N* in the case of homogeneous eigenvector components, but remains finite as 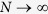 if activity is concentrated on a finite number of nodes. Numerical evidence has also been provided for the relation of *localization to RR effects, slowing down the dynamics to follow power-laws*^17^.

**Figure 14:**
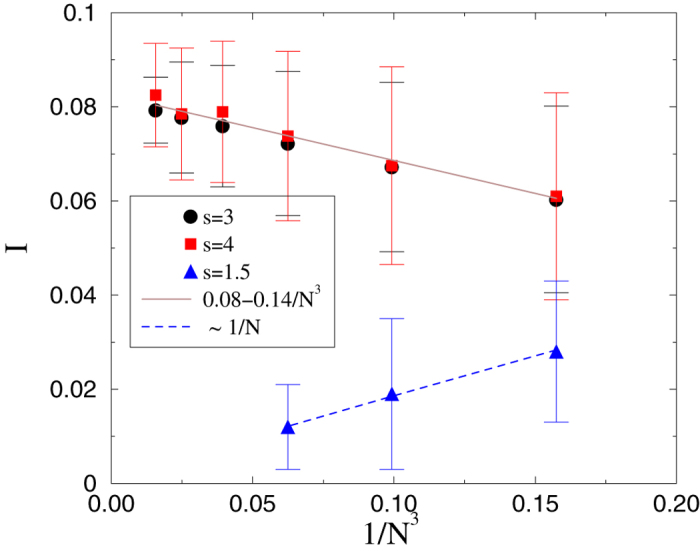
Finite size scaling of the inverse participation ratio in weakly coupled HMN2d models, with maximum levels *l*_*max*_ = 4, 5, ..9. The *s* = 3 (bullets) and *s* = 4 (boxes) results suggest localization (finite *I*) in the infinite-size limit. Lines are power-law fits to the data. For *s* = 1.5, corresponding to the symmetrized, small-world network (model-6 of^38^) no evidence of localization is seen.

We study localization of the SIS in the HMN2d models introduced in the previous section*s. We analyze the eigenvectors of *B*_*ij*_ for *b* = 2 (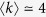), varying *s*, using system sizes ranging from *N* = 256 to *N* = 262144. In particular, we performed a FSS scaling study of the IPR in these systems. First we determined the behavior on the small world network of^38^ corresponding to *s* = 3/2. As one can see in Fig. 14, the localization at small sizes disappears as 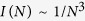. By contrast, for *s* = 3 and *s* = 4, the graphs are finite-dimensional, and the IPR increases with *N*, tending to a finite limiting value, suggesting localization.

**Figure 15:**
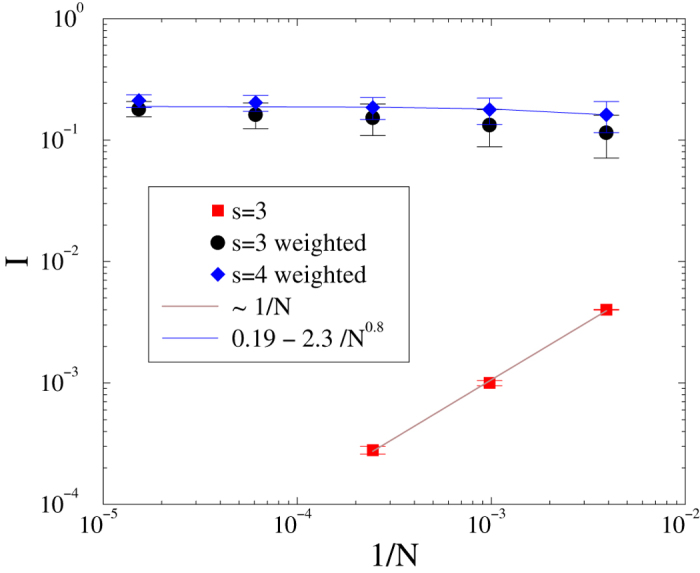
Finite size scaling of the inverse participation ratio in HMN2d models with higher average degree (〈*k*〉 ≃ 50) for maximum levels: *l*_*max*_ = 4, 5, 6, 7, 8. Bullets: *s* = 3 with uniform randomly distributed weights; boxes: without weights. Diamonds: *s* = 4 with randomly distributed weights. Lines show power-law fits to the data. In the unweighted case, no localization effect can be seen, and *I* decays linearly with *N*.

One might question the relevance of network models with small connectivity to mammalian brains, in which 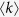 is on the order of 10^3^. To answer this we study *s* ≤ 4 models with higher connectivity, i.e., 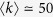. As Fig. 15 shows, the sign of localization, which is weak but nonzero for 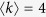, now disappears. Next, we add random weights *w*_*ij*_, distributed uniformly over the interval (0, 1), to the links of the networks. A consequence of this explicit disorder is localization even in highly connected networks, as shown in Fig. 15. This result is in agreement with the expectation of limited sustained activity in brain networks^38^, meaning that the link disorder prevents over-excitation of a network of high connectivity.

**Figure 16:**
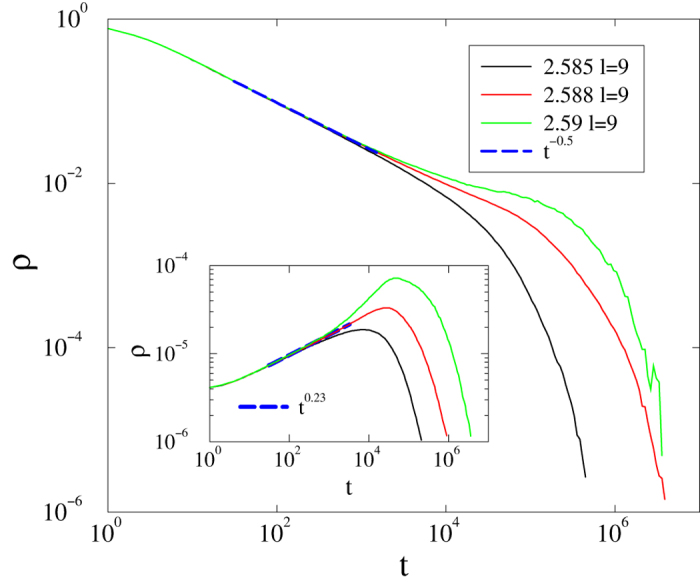
Evolution of the activity in the weighted CP on HMN2d networks with *s* = 4, *l* = 9, and *k* = 〈50〉, for *λ* = 2.585, 2.588, and 2.59 (lower to upper). Main plot: decay in case of a fully active initial state. Inset: growth of activity starting from a single active node for the same values of *λ*. Dashed lines are power-law fits for 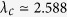.

Localization suggests rare-region effects, thus a dynamic GP. Nevertheless, simulations of the CP on such networks do not show extended regions of power-laws for *s* ≤ 4, but rather a nontrivial (non-mean-field) continuous phase transition (Fig. 16). Decay simulations for *l* = 9 yield 
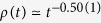
 at 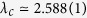, albeit with a cutoff due to the finite system size. This agrees with our result for 1D BB networks with *s* = 3^15^. Spreading simulations starting from single, randomly placed seed result in the survival probability scaling at this critical point: 
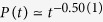
 (that is, the symmetry *α* = *δ* holds to within uncertainty). Here the density increases initially as 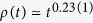. One can understand these results, noting that the localization values are rather small, *I* ~ 0.08, so that RR effects are too weak to generate observable GP effects in the dynamics.

Naturally, for *s* > 4 networks, where already the topological disorder is strong enough to create a GP, inhomogeneities in the interaction strengths amplify the RR effects. This induces GPs even for larger values of 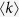, as well as in the threshold models. Further studies of disorder effects are under way.

## Conclusions

We investigate the dynamical behavior of spreading models on hierarchical modular networks embedded in two-dimensional space, with long links whose probability decays as a power-law with distance. This corresponds to an exponentially decreasing connection probability, as a function of the level in the hierarchical construction. The aim of this study is to understand the effects of intrinsic and topological disorder, in particular, extended critical regions in the parameter space, without any (self) tuning mechanism.

If we eliminate the underlying lattice structure we observe power-law dynamics for networks near the percolation threshold, when the effective dimension is finite. However, size-independent power-laws, corresponding to GPs, are only seen if we have directed links. Since connectivity of these structures is not guaranteed, we also study random hierarchical trees, with full connectivity. GPs are observed upon averaging over many independent network realizations. The relation to brain networks can be envisaged in the large-size limit, if we regard independent realizations as (almost decoupled) sub-modules of the entire brain.

When we ensure connectivity via short edges on the lowest level, we find GPs for rapidly decaying long-link probabilities. Both of these network assumptions are in accordance with empirical brain networks. Above the GP, at the critical point, we find mean-field-like decay of activity, in agreement with results on the CP with quenched disorder on higher-dimensional, regular lattices. We have also shown that bursty behavior arises naturally in the GPs, due to autocorrelations which decay via a power-law.

We perform a quenched mean-field study of the SIS model on these networks; in the SIS, nodes are connected symmetrically. Finite size scaling of the inverse participation ratio suggests localization on large-world networks and de-localization on small world structures. However, when we add intrinsic weight disorder, localization can be seen even on small-world networks. Weight disorder is again to be expected in real brain networks, since the strength of couplings varies over many orders of magnitude. Despite this, we saw no GP effects in the dynamics of weighted CPs with *s* = 4. Instead, we find a nontrivial critical scaling, as has already been observed in other networks. The possibility of a narrow GP in this case is an open issue.

In conclusion, we believe our synthetic HMN2ds are closer to experimental brain networks than previously proposed models, and find numerical evidence for GPs in extended phases in simple models with spreading dynamics. Although we eliminate any self-tuning mechanisms, we still find nontrivial slow dynamics as well as localization of activity, which is crucial for understanding real brain network data.

## Additional Information

**How to cite this article**: Ódor, G. *et al.* Griffiths phases and localization in hierarchical modular networks. *Sci. Rep.* **5**, 14451; doi: 10.1038/srep14451 (2015).

## Supplementary Material

Supplementary Information
