# Supplementary material for: Griffiths phases and localization in hierarchical modular networks
Source: Sci Rep. 2015 Sep 24;5:14451. doi: 10.1038/srep14451 (PMC4585858; doi:10.1038/srep14451)
Supplement: Supplementary Information [file srep14451-s1.pdf]

Supplementary information
Griffiths phases and localization in hierarchical modular networks

Géza Ódor[1], Ronald Dickman[2] and Gergely Ódor[3]
[1] *MTA-MFA-EK Research Institute for Technical Physics and Materials Science, H-1121 Budapest, P.O. Box 49, Hungary*
[2] *Departamento de Fisica and National Institute of Science and Technology of Complex Systems, ICEx, Universidade Federal de Minas Gerais, Caixa Postal 702, 30161-970, Belo Horizonte - Minas Gerais, Brazil*
[3] *Massachusetts Institute of Technology, 77 Massachusetts Avenue Cambridge, MA 02139-4307, USA*

Although the set of nodes is isomorphic to a square lattice of $N = 2^L \times 2^L$ sites, we shall label the nodes not by their Cartesian coordinates but rather using a base-4 notation. At level l, the full set of 4^l nodes is divided into four quadrants labeled 0, 1, 2, and 3, proceeding counterclockwise from the lower left. At level $l-1$, each of the quadrants is similarly divided into four subquadrants, labeled in the same manner. Division into subquadrants continues all the way down to level 1, in which the elements are individual nodes. A given site can be specified by the labels of the quadrant, subquadrant, etc. to which it belongs. Denoting the labels at levels 1, 2,..., L, by $g_1, ..., g_L$, the address of a site is $n = \sum_{l=1}^{L} g_l \, 4^{l-1}$ (see Fig. S1).

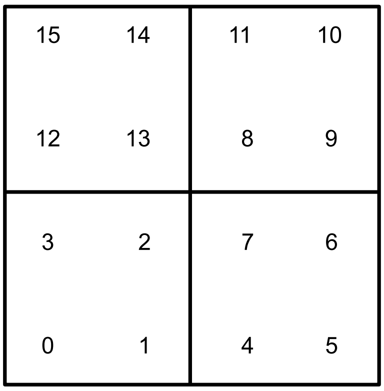

Supplementary Figure S 1: Definition of node labels for a network with $L = 2$

Consider a set of four nodes, 0, 1, 2, and 3. There are six possible links between them: (0,1), (0,2), (0,3), (1,2), (1,3), and (2,3). A tree with four nodes has exactly three links. Of the $\binom{6}{3} = 20$ subsets of three links, sixteen yield a connected tree [for example: {(0,1), (1,2), (2,3)}], while four correspond to a cycle of three nodes, leaving the fourth node isolated [example: {(1,2), (1,3), (2,3)}]. To construct a tree of four nodes, we choose one of the sixteen link sets $\mathcal{B}_i$ at random.

A hierarchical tree is constructed by first linking the four quadrants via three edges. The link set $\mathcal{B}_i$ is chosen at random. Then, for each link, we choose sites at random within each of the two quadrants connected by the link, to serve as the connected nodes. Now we repeat this process within each of the subquadrants, and so on, until we reach the basic modules of four sites. The latter are again connected by sets of three links, chosen independently from the basic collection of sixteen link sets. At the end, each basic module is connected internally, and to a module at the next level, etc., so that we have a connected graph of N nodes and $N-1$ edges. Although we have chosen to begin the construction at level L, we could equally have begun at level 1, or some intermediate level: the choices of link sets and nodes at the various levels are mutually independent. These steps are illustrated in the Figs. S2, S3, S4.

At level 1 the number of links per node is 3/4; at level j, there are 4^{L-j} blocks connected via $4^{L-j} - 1$ links. The number of links per node at this level is therefore $(4^{L-j} - 1)/4^L \simeq 1/4^j$. Let p_j denote the probability that a randomly chosen edge linking blocks at level j be present. At level 1, links connect nodes within four-site modules. Since there are six possible links, and since just three are present within each module, we have $p_1 = 1/2$. At level 2, a link connects nodes within a 16-node module; at this level a node is linked to one of the 12 nodes outside its basic 4-site module. There are $(16 \cdot 12)/2 = 96$ possible links, and since only three are present, $p_2 = 1/32$. At level j, an

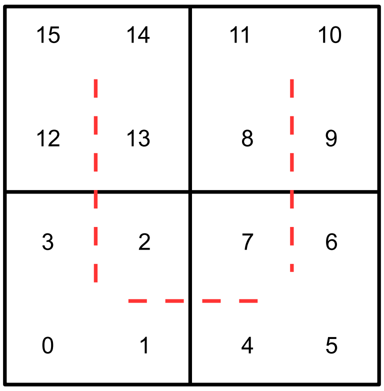

Supplementary Figure S 2: First step in constructing a network with $L = 2$: define the link set at level 2.

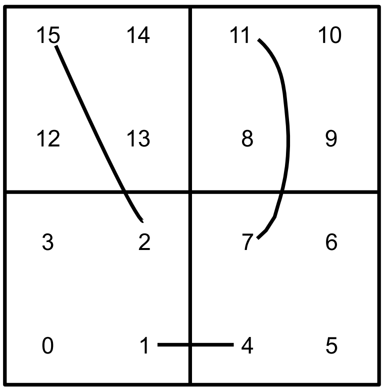

Supplementary Figure S 3: Second step: choose nodes consistent with link set at level 2. At the next step (not shown) we choose link sets for each of the four-site modules.

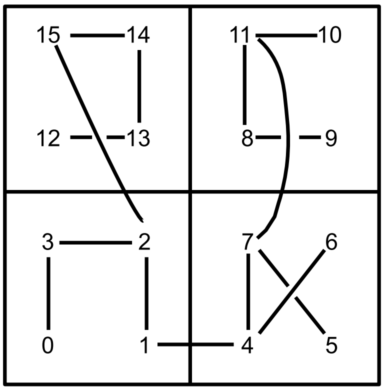

Supplementary Figure S 4: Final step: choose nodes at level 1 consistent with the corresponding link sets.

edge links nodes within a module containing 4^j nodes. The number of possible links at this level is $4^j(3 \cdot 4^{j-1})/2$, so that $p_j = 1/2^{4j-3}$, and, in general, $p_j/p_{j-1} = 1/16$. Since a tree represents a minimally connected structure, p_j cannot decay faster than this rate, in any connected hierarchical network based on four-node modules.

Although our principal interest is in random trees, as described above, we may also consider *regular* trees; these are constructed by using the same link set at each level. The resulting structure turns out to have infinite topological dimension, as discussed in Section "Dimension measurements".
